# Sound-Evoked Responses in the Vestibulo-Ocular Reflex Pathways of Rats

**DOI:** 10.3389/fnins.2021.741571

**Published:** 2021-10-14

**Authors:** Tianwen Chen, Jun Huang, Yue Yu, Xuehui Tang, Chunming Zhang, Youguo Xu, Alberto Arteaga, Jerome Allison, William Mustain, Matthew C. Donald, Tracy Rappai, Michael Zhang, Wu Zhou, Hong Zhu

**Affiliations:** ^1^Department of Otolaryngology-Head and Neck Surgery, University of Mississippi Medical Center, Jackson, MS, United States; ^2^Department of Otolaryngology, First Affiliated Hospital, Shanxi Medical University, Taiyuan, China; ^3^Department of Neurobiology and Anatomical Sciences, University of Mississippi Medical Center, Jackson, MS, United States; ^4^School of Medicine, University of Mississippi Medical Center, Jackson, MS, United States; ^5^Summer Undergraduate Research Program, University of Mississippi Medical Center, Jackson, MS, United States; ^6^Department of Neurology, University of Mississippi Medical Center, Jackson, MS, United States

**Keywords:** vestibular-evoked myogenic potential (VEMP), single unit recording, vestibulo ocular reflex, otolith, canal, eye movement, abducens nucleus

## Abstract

Vestibular evoked myogenic potentials (VEMP) have been used to assess otolith function in clinics worldwide. However, there are accumulating evidence suggesting that the clinically used sound stimuli activate not only the otolith afferents, but also the canal afferents, indicating canal contributions to the VEMPs. To better understand the neural mechanisms underlying the VEMPs and develop discriminative VEMP protocols, we further examined sound-evoked responses of the vestibular nucleus neurons and the abducens neurons, which have the interneurons and motoneurons of the vestibulo-ocular reflex (VOR) pathways. Air-conducted clicks (50–80 dB SL re ABR threshold, 0.1 ms duration) or tone bursts (60–80 dB SL, 125–4,000 Hz, 8 ms plateau, 1 ms rise/fall) were delivered to the ears of Sprague-Dawley or Long-Evans rats. Among 425 vestibular nucleus neurons recorded in anesthetized rats and 18 abducens neurons recorded in awake rats, sound activated 35.9% of the vestibular neurons that increased discharge rates for ipsilateral head rotation (Type I neuron), 15.7% of the vestibular neurons that increased discharge rates for contralateral head rotation (Type II neuron), 57.2% of the vestibular neurons that did not change discharge rates during head rotation (non-canal neuron), and 38.9% of the abducens neurons. Sound sensitive vestibular nucleus neurons and abducens neurons exhibited characteristic tuning curves that reflected convergence of canal and otolith inputs in the VOR pathways. Tone bursts also evoked well-defined eye movements that increased with tone intensity and duration and exhibited peak frequency of ∼1,500 Hz. For the left eye, tone bursts evoked upward/rightward eye movements for ipsilateral stimulation, and downward/leftward eye movements for contralateral stimulation. These results demonstrate that sound stimulation results in activation of the canal and otolith VOR pathways that can be measured by eye tracking devices to develop discriminative tests of vestibular function in animal models and in humans.

## Introduction

Since the discovery of the vestibular-evoked myogenic potentials (VEMPs) (Colebatch and Halmagyi, 1992), there has been a rapid growth of VEMP research and investigators worldwide have used the VEMPs to characterize a variety of vestibulopathies, including Tullio/superior canal dehiscence syndrome, vestibular neuritis, Ménière’s disease, and vestibular schwannoma (Minor et al., 1998; Rauch, 2006; Rosengren et al., 2010). Although the VEMPs have been widely used, important issues on the neural basis remain to be elucidated in order to develop more discriminative VEMP protocols and interpretative guidelines. Because early animal studies showed that loud sound primarily activates the otolith afferents (Murofushi et al., 1995; Murofushi and Curthoys, 1997), the VEMPs are presently used to test otolith function. However, there are studies suggesting that loud sound also activates other vestibular end organs. For example, Young et al. (1977) and Carey et al. (2004) reported that long duration tones activated both the canals and the otoliths in monkeys and chinchillas, respectively. We further found that the clinical VEMP stimuli activated both the canal and otolith afferents in rats (Zhu et al., 2011b, 2014). Consistent with these single unit recording results, our intra-axonal labeling studies provided anatomical evidence that sound sensitive afferents innervate horizontal and anterior cristae as well as saccular and utricular macule (Zhu et al., 2014).

The goal of the present study was to extend the studies of sound activation of the vestibular afferents to examine sound activation of the vestibular nucleus neurons and the abducens neurons, which mediate the ocular VEMP (oVEMP) recorded over the extraocular muscles (Wilson and Schor, 1999; Uchino et al., 2005; Uchino and Kushiro, 2011). Furthermore, we employed a video-based eye tracker to examine tone burst-evoked eye movements in awake rats. These results show that sound activation of the vestibular afferents are processed by the vestibular nucleus neurons, which then activate the VOR motoneurons to generate sound-evoked eye movements. This knowledge not only provides insight into neural mechanisms underlying the VEMPs, but also provides evidence showing the potentials of sound-evoked eye movement as a test of vestibular function in animal models and in humans.

## Materials and Methods

Adult male Sprague-Dawley (SD) rats weighing 250–350 g (Harlan, Indianapolis, IN, United States) were used in the experiments of recording vestibular nucleus neurons in anesthetized condition. Adult female Long-Evens (LE) rats weighing 175–225 g (Harlan, Indianapolis, IN, United States) were used in the experiments of single unit recording of the abducens neurons and recording of eye movements in awake condition. The abducens recording and sound-evoked eye movement experiments were initiated after completion of the vestibular nucleus recording experiments. These experiments need to be performed in a different strain of rats (i.e., Long-Evans rats) because we found out that SD rats were albino and their pupils could not be tracked by the ISCAN eye tracker. All procedures were approved by the Institutional Animal Care and Use Committee at University of Mississippi Medical Center.

### Single Unit Recording of the Vestibular Nucleus Neurons

#### Surgeries

Sound-evoked responses in vestibular nuclei were first studied in anesthetized animals by Murofushi et al. (1996). To be comparable to the literature, we performed vestibular nucleus neuron recordings in anesthesia rats (sodium pentobarbital, 50 mg/kg, i.p.). The animals’ core body temperature was monitored and maintained at 36–37°C with a heating pad (Frederick Haer & Company, Bowdoinham, ME, United States). A midline dorsal cranial skin incision was made and soft tissues were cleared to expose skull suture landmarks bregma and lambda. A small stainless steel cylinder was cemented on the skull by dental acrylic for stabilization of the rat’s head during recording experiments. The head was stabilized on a stereotaxic frame with the head holder by a Kopf stereotaxic carriage (David Kopf Instruments, Tujunga, CA, United States). A burr-hole was made in the skull over the coordinates corresponding to the vestibular nuclei (From Lamda, L 1.5–2.0 mm, AP 3.6–4.6 mm, D 7–8 mm, Paxinos and Watson, 1998). A microelectrode (Sutter Instruments, Novato, CA, United States) filled with 3M sodium chloride (5 MΩ) that was mounted on a microdrive was then advanced into the vestibular nuclei. Signals were amplified and filtered by a MNAP system (Plexon Inc., Dallas, TX, United States). Once a unit was isolated, at least 30 s background discharge activity was recorded. Then the unit’s responses to head rotation and sound stimulation were recorded.

#### Vestibular Stimulation

The animal’s body was restrained in a nylon jacket and stabilized in the stereotaxic instrument. The stereotaxic instrument was mounted on a custom-made rotation device that allowed us to deliver head rotations. In order to stimulate horizontal and vertical canals, the head was tilted 30° nose down and 15° left ear down (Estes et al., 1975; Daunicht and Pellionisz, 1987; Blanks and Torigoe, 1989) and subjected to sinusoidal earth-horizontal rotations (1 Hz, 10°, peak velocity of 62.8 deg/s). Single unit data along with horizontal and vertical head position signals were recorded for at least 10 cycles.

#### Sound Stimulation

Sound was generated by a MA3 stereo microphone amplifier (DT system, Tucker-Davis Technologies, Alachua, FL, United States) and delivered via an insert ear phone (ER-3A). A hard plastic infant tip adapter was placed at the end of a sound conducting tubing and placed into a speculum which was sealed in the ear canal. Before and after the speculum was inserted, the ear canal was checked with an otoscope to ensure that it was patent. To ensure comparability of results between animals, sound intensity was referred to the threshold of the auditory brainstem response (ABR) of individual animals. ABR threshold was determined for each rat using stainless steel subdermal needle electrodes placed at the vertex (active), behind the stimulated ear (reference) and in the hind leg (ground) (Simpson et al., 1985). Differentially recorded signals were amplified (100,000×), filtered (100 Hz–3 kHz) and digitized at 20 kHz over a 15 ms epoch (ICS Chartr EP 200 evoked potential assessment device, GN Otometrics, Taastrup, Denmark). Stimulus was a 0.1 ms click of alternating polarity, delivered through the earphone at a rate of 25.1/s. ABR threshold was determined by averaging 2,000 responses. Animals with elevated thresholds were excluded to eliminate the effects of a possible conductive hearing loss.

Air-conducted clicks (50–80 dB re ABR threshold, 0.1 ms duration, rarefaction or condensation) or tone bursts with various frequencies and intensities (125–4,000 Hz; 8 ms plateau, 1 ms rise/fall; 60–80 dB SL re ABR threshold) were delivered randomly at a rate of 5 Hz to the ear ipsilateral to the recording site. Typically, 150 trials were obtained for each condition (100 ms pre-stimulus to 100 ms post-stimulus).

### Single Unit Recording of the Abducens Neurons

Under general anesthesia, a burr hole (∼3 mm) was made in the skull over the coordinates corresponding to the abducens nucleus (From Lamda, L 0–0.5 mm, AP 3.6–4.0 mm, D 8–8.5 mm, Paxinos and Watson, 1998) and the dura was removed. The craniotomy was closed by bone wax and protected by a recording chamber secured to the skull by dental acrylic. The chamber was covered by a cap. Rats were given at least 7 days to allow a full recovery. Because a pilot study showed that few abducens neurons were activated by sound stimulation in anesthetized rats, in this study, we performed single unit recordings of the abducens neurons in awake LE rats. Each rat was briefly anesthetized with isoflurane and the head was stabilized on a stereotaxic frame via the head holder. To avoid bleeding due to hitting the sinus, the head was positioned 18° downward with respect to the horizontal arm of the stereotaxic instrument. A microelectrode was advanced downward by a microdrive to search for abducens neurons. Once a neuron was isolated, its responses to spontaneous eye movements and eye movements evoked by passive head rotations were recorded. Then its responses to air-conduced clicks or tone bursts delivered to the ipsilateral and contralateral ears were recorded. While the atlas coordinates were used as the guide to search for abducens neurons, the final identification of abducens neurons was based on their eye movement sensitivity and histological reconstruction.

### Eye Movement Recording

Female LE rats were used to study sound-evoked eye movements since their pupils are better tracked for eye movement recording and they have been shown to tolerate the restraint method well (Quinn et al., 1998). The rat eye movement was recorded using a video-based eye tracker (ISCAN ETS-200, ISCAN, Burlington, MA, United States) as described in our previous study (Stewart et al., 2016). Briefly, a rat’s head and body was secured on a stereotaxic instrument that was mounted on a servo-controlled rotator. An infrared camera equipped with a zoom lens (Computar TV Zoom Lens, Computar Optics Group, Japan) was attached to the platform of the rotator and was focused on the eye. The rat’s left eye was illuminated by a standard ISCAN multiple infrared LED illuminator attached to the camera mount on a flexible arm to produce a reference corneal reflection (CR). The ISCAN system tracks the centers of the pupil and CR and provides real-time signals related to pupil position and CR position, which were digitized and sampled at 1 kHz with head position signals by a CED Power 1401 system (Cambridge Electronics Devices, Cambridge, United Kingdom). Calibration was achieved by rotating the camera by ±10° around the vertical axis of the turntable.

### Histology

After completion of the single unit recording experiments, a dye deposit was made in the recording site by injecting of 7% fast green with a current of 7 μA for 10 min. Brain sections (45 μm) were cut and stained with cresyl violet to recover the recording sites.

### Data Acquisition and Data Analysis

Extracellular voltage signals were filtered and sampled by a CED Power 1401 system (Cambridge Electronics Devices, Cambridge, United Kingdom) at 20 kHz with 16-bit resolution and a temporal resolution of 0.01 ms. Signals of horizontal and vertical head positions and sound stimulation were sampled at 1 kHz. These signals were stored on a hard disk for offline analyses. Data analysis was performed on PC workstations using Spike 2 (Cambridge Electronics Devices, Cambridge, United Kingdom), MatLab (The MathWorks, Inc. Natick, MA, United States) and SigmaPlot (Systat Software, Inc., San Jose, CA, United States). Coefficient of variation (CV) of interspike intervals was used to assess regularity of spontaneous discharge of vestibular nucleus neurons. For vestibular afferents in rodents, CV has been shown to be dependent on firing rate and CV^∗^ has been used to normalize CV (Lasker et al., 2008). For vestibular nucleus neurons, however, the relationship between CV and firing rate has not been established. Thus, we did not attempt to adopt the CV^∗^ approach to normalize CV in vestibular nucleus neurons. Head motion signals and neuronal firing rate data were averaged over multiple sinusoidal cycles. A fast Fourier transform (FFT) analysis was performed to compute the gain and phase of the unit with respect to the fundamental frequency. Gain and phase relative to head velocity were calculated at the fundamental stimulus frequency (∼0.5 Hz). Neurons that were excited by ipsilateral head rotation were classified as Type I neurons. Vestibular nucleus neurons that were excited by contralateral head rotation were classified as Type II neurons. This classification was adopted from Scudder and Fuchs (1992). Because we were unable to test type-II neurons for pitch rotations, type-II neurons likely include both true-Type II neurons and vertical canal neurons. Neurons that were not modulated during head rotation likely received inputs from otolith afferents and were classified as non-canal neurons.

**FIGURE 1:**
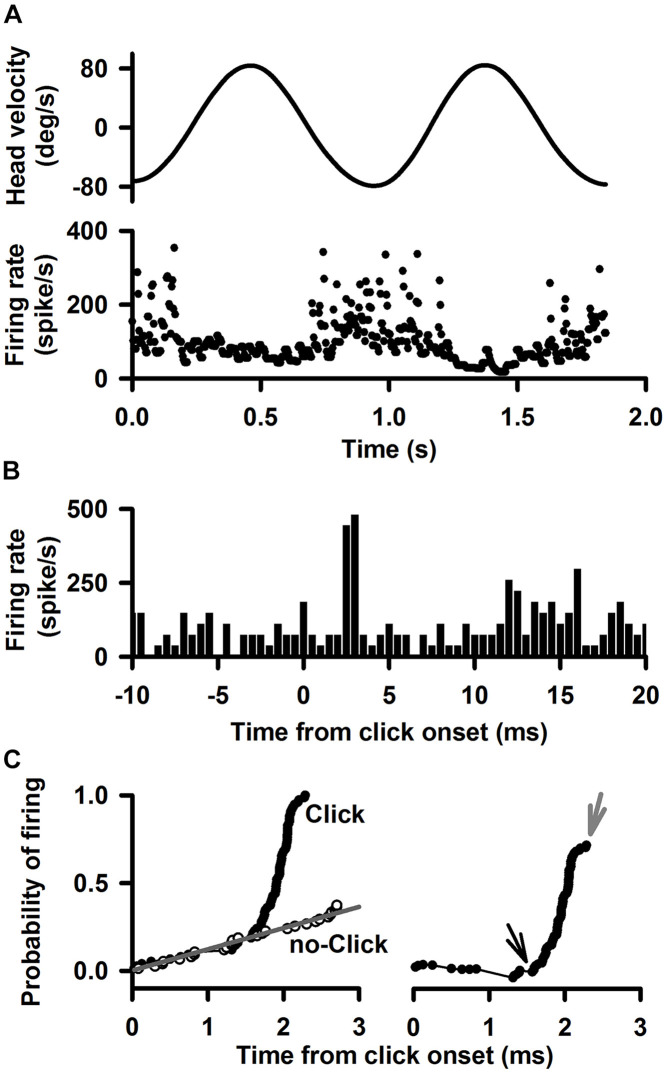
A representative Type I neuron’s responses to clicks (0.1 ms, rarefaction, 50–80 dB SL, 0 dB is referred to the threshold of the ABR) delivered to the ipsilateral ear. **(A)** Upper trace: head velocity. Lower trace: firing rate of the neuron in response to 1 Hz horizontal rotation. **(B)** Peristimulus histograms of click-evoked responses at 80 dB SL. Bin size is 0.5 ms. **(C)** Quantitative measurement of the time course of the click-evoked excitatory response. *Left panel* probability of evoking a spike. The black curve labeled “click” plots the probability that the neuron fired as a function of time after click onset. The gray curve labeled “no click” plots the probability that the neuron fired as a function of time during spontaneous discharging. *Right panel* Probability of evoking a spike as a function of time, obtained by subtracting the regression line (no-click, gray) in the upper panel from the data for “click” case. Amplitude of evoked-response is defined by cumulative probability of evoking a spike (CPE), which is estimated as peak probability (gray arrow) minus the baseline probability (black arrow). Latency of click-evoked response is defined as the onset of the sharp increase in firing probability (black arrow).

Sound sensitivity of the vestibular nucleus neurons and abducens neurons was quantified by computing the cumulative probability of evoking a spike (CPE) (Broussard and Lisberger, 1992; Broussard et al., 1995; Zhu et al., 2011b, 2014) which was able to accurately compute the latency and amplitude of the sound-evoked responses and to quantitatively assess relative sound sensitivity among different neurons. To compute the CPE, first, the latency between the click or tone burst and the foot of the first action potential after sound onset was measured for 150 sound stimuli. Then the latencies were arranged in ascending order and were paired with an ascending series of probabilities ranging in equal increments from 1/150 to 1.0. To estimate the time course of the probability of firing after sound onset, probability of firing was plotted as a function of the time after sound onset (“click,” Figure 1C, left panel). To take into account the probability that the neuron would have fired in the absence of sound, the same analysis was performed beginning 30 ms before sound onset (“no-click,” Figure 1C, left panel). Linear regression was used to fit a line to the “no-click” data and the Y-value of the line was subtracted from each “click” value to yield the probability of evoking a spike as a function of time (Figure 1C, right panel). The latency of the sound-evoked response was defined as the onset of the abrupt increase in firing probability (Figure 1C, right panel, the point of divergence between the line and the data points in the Click condition). The amplitude of the neuron response was measured by the height of the rapid change in firing probability. A neuron was classified as sound sensitive if its CPE was larger than 0.1, i.e., clicks or tones increased firing probability by 0.1.

### Statistical Analysis

Statistical analyses were performed using SigmaPlot. Differences among experimental groups were analyzed by one-way or two-way ANOVAs. Differences between two experimental groups were analyzed by *t*-tests or paired *t* tests. *P* values of less than 0.05 were considered statistically significant. Mean values +SE are presented.

## Results

### Sound-Evoked Responses of the Vestibular Nucleus Neurons

A total of 425 vestibular nucleus neurons were recorded from 62 rats (Table 1). Figure 1 shows typical responses to click of a Type I vestibular nucleus neuron, which increased its firing rate to ipsilateral head rotation (1 Hz) with a gain of 0.45 (spike/s)/(deg/s) and a phase lag of 3.5° with respect to the ipsilateral head velocity (Figure 1A). The peristimulus histograms exhibit short-latency excitatory responses to clicks of 80 dB SL (Figure 1B). The cumulative probability of evoking a spike, i.e., CPE, as a function of time was computed to measure latency and amplitude of the sound-evoked responses (Figure 1C). Latency of the sound-evoked response was defined as the onset of the sharp increase in firing probability (Figure 1C, right panel, black arrow). Amplitude of the sound-evoked response was estimated as the difference between the peak probability (gray arrow) and the baseline probability (black arrow). This Type I vestibular neuron has a latency of 1.36 ms and an amplitude of 0.81 for clicks at 80 dB SL.

**TABLE 1:** Summary of the responses of vestibular nucleus neurons to clicks or tone bursts.

	Clicks			Tones		
	Type I	Type II	Non-canal	Type I	Type II	Non-canal
SS	42	7	37	24	7	50
NSS	64	54	13	54	21	52
Total	106	61	50	78	28	102
% of SS	39.6%	11.5%	74%	30.8%	25%	49%

*Data are presented as number of neurons or and percentage of sound sensitive neuron. Neurons that were excited by ipsilateral earth-horizontal rotations were classified as Type I neurons. Neurons that were excited by contralateral earth-horizontal rotations were classified as Type II neurons. Neurons that were not modulated during earth-horizontal rotation were classified as non-canal neurons. SS, sound sensitive neuron; NSS, non-sound sensitive neurons. A SS neuron is defined as CPE values are above 0.1. See text for statistical analysis.*

#### Click-Evoked Responses of Vestibular Nucleus Neurons

**FIGURE 2:**
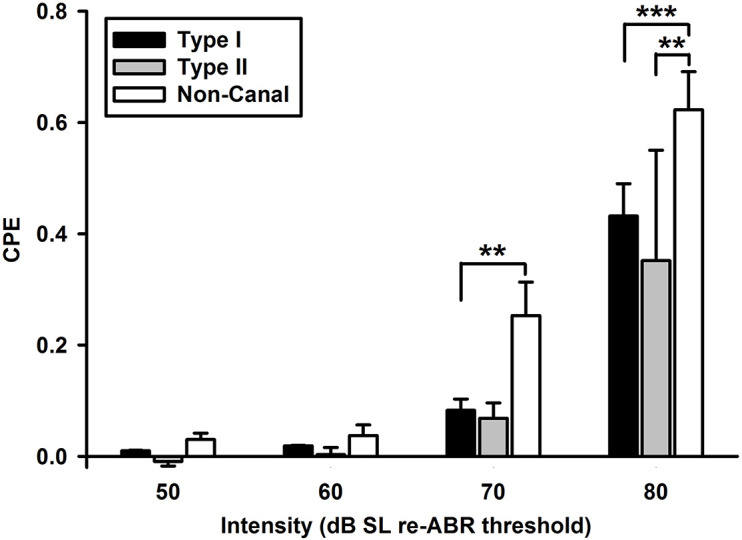
Click-evoked responses of vestibular nucleus neurons (Type I, Type II, and non-canal neurons) as a function of click intensity. All types of neurons showed increased responses with higher sound intensities. The non-canal neurons had larger click-evoked responses than Type I and Type II neurons. ***P < 0.001, **P < 0.01.

Click-evoked responses were studied in 217 of the 425 vestibular nucleus neurons. There were more sound sensitive Type I neurons than Type II neurons (39.6 vs. 11.5%, Chi-square test, *P* < 0.001) and more sound sensitive non-canal neurons (74%) than the Type I or Type II neurons (Chi-square test, *P* < 0.032 and *P* < 0.001, respectively) (Table 1). A two-way ANOVA of 35 vestibular nucleus neurons (17 Type I, 3 Type II, and 15 non-canal) that were tested with four intensity levels (50–80 dB SL) revealed significant effects of click intensity (*P* < 0.001, *F*_3_,_139_ = 40.3) and neuron type (*P* < 0.001, *F*_2_,_139_ = 8.6) on CPE but no significant interaction between the two factors (*F*_6_,_139_ = 1.8, *P* = 0.097). *Post hoc* analysis (Holm-Sidak) revealed that all types of neurons showed increased responses with higher sound intensities (*P* < 0.05), but there was no significant difference between 50 and 60 dB conditions (*P* = 0.846; Figure 2). The threshold to activate the non-canal neurons was 70 dB SL while the threshold to activate the canal sensitive neurons was 80 dB SL. The non-canal neurons had larger CPEs than the Type I (*T* = 3.77, *P* < 0.001) and Type II neurons (*T* = 2.80, *P* = 0.012). There was no significant difference in CPE amplitudes between Type I and Type II neurons (*T* = 0.69, *P* = 0.49).

**FIGURE 3:**
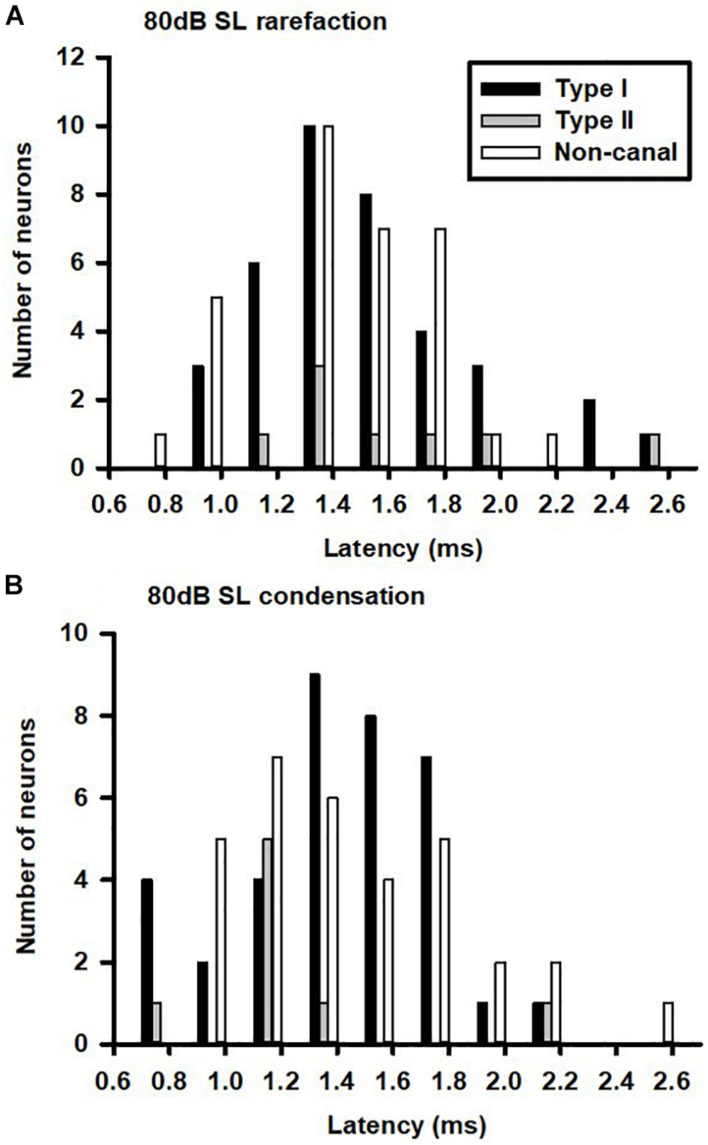
Latency distributions of click-evoked responses in vestibular nucleus neurons. **(A)** Rarefaction and **(B)** condensation.

The vestibular nucleus neurons responded to both rarefaction and condensation clicks. Click polarity exhibited no significant effects on the CPE amplitudes of the Type I, Type II, and non-canal neurons. Figures 3A,B show the latency distributions for sound sensitive vestibular nucleus neurons. Averaged response latencies to rarefaction clicks and condensation clicks were 1.43 ± 0.04 and 1.37 ± 0.04 ms, respectively. Overall, there was no significant difference in the response latencies between the two conditions (Paired-*t* test, *t* = 1.44, *P* > 0.05). One way ANOVA analysis showed no significant difference in response latency among different types of neurons for rarefaction and condensation clicks.

**FIGURE 4:**
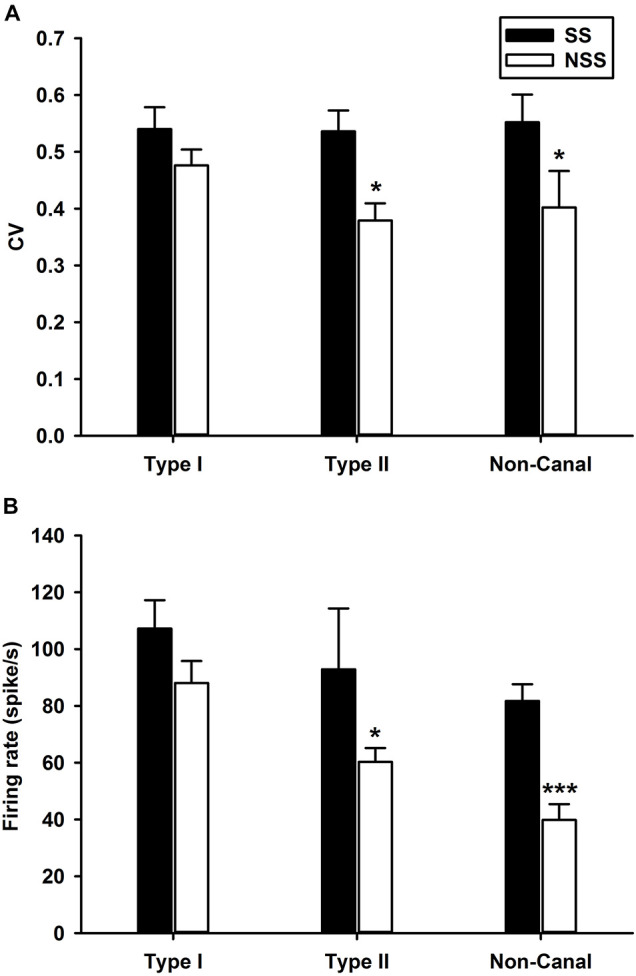
Comparisons of spontaneous firing rates and regularity (CV) between sound sensitive (SS) and non-sound sensitive (NSS) vestibular nucleus neurons. **(A)** CV. The SS Type II and non-canal neurons were more irregular than NSS neurons. **(B)** Spontaneous firing rates. The SS Type II and non-canal neurons had higher spontaneous firing rates than NSS neurons. ***P < 0.001, *P < 0.05.

**FIGURE 5:**
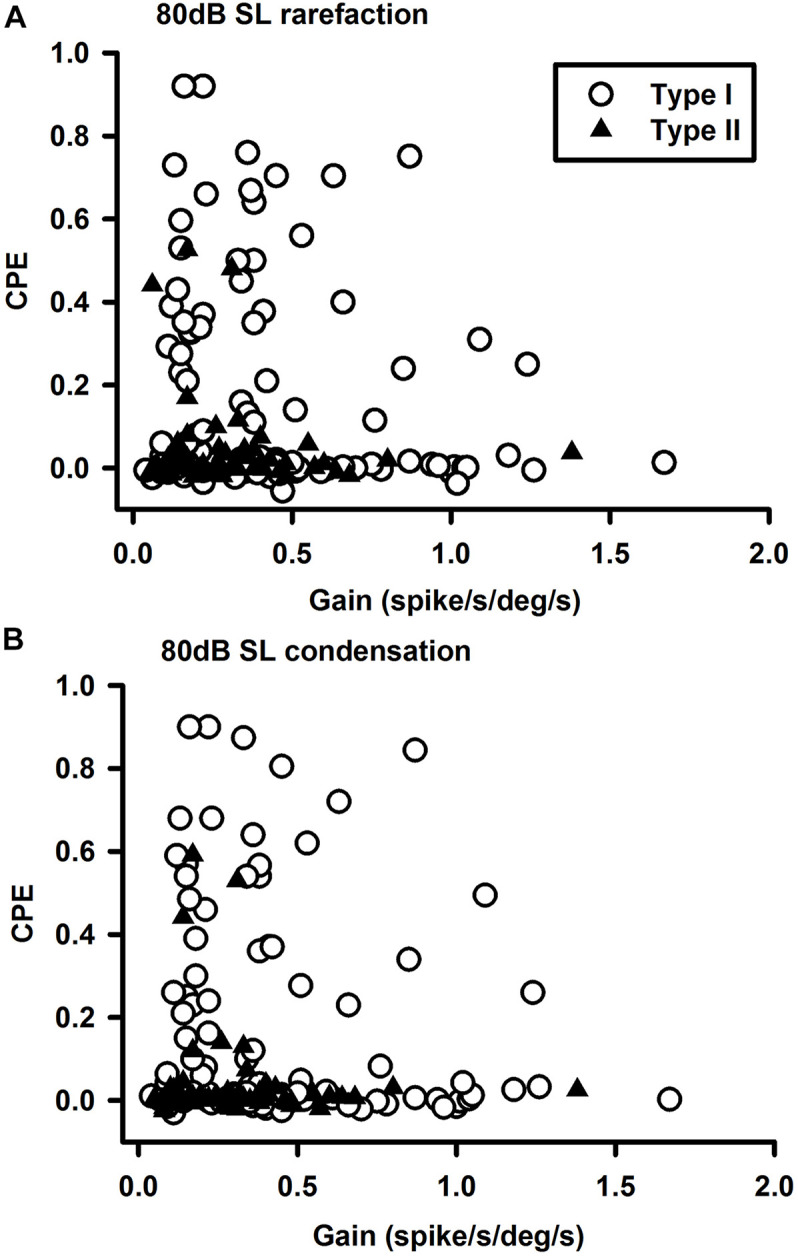
Relationships between vestibular nucleus neuron’s sensitivities to clicks and head rotation at 80 dB SL. **(A)** Rarefaction and **(B)** condensation. The sound sensitivities (CPEs) of the Type I neurons were inversely correlated with their sensitivities to head rotations (gains).

The sound sensitive neurons (SS) and the non-sound sensitive neurons (NSS) exhibited significant differences in spontaneous firing rates and regularity (Figure 4). For Type II neurons (*t*-test, *P* < 0.05, *T* = 1.703) and non-canal neurons (*t*-test, *P* < 0.05, *T* = 1.72), CV of the SS neurons were significantly larger than CV of the NSS neurons (Figure 4A). While the SS Type I neurons exhibited larger CV than the NSS type I neurons, the difference did not reach significance (*t*-test, *P* = 0.09, *T* = 1.359). It is important to note that in this analysis, CV of the vestibular nucleus neurons is not normalized with respect to firing rate. When future studies establish the relationship between CV and firing rate for vestibular nucleus neurons, this issue needs to be revisited. The spontaneous firing rates for sound sensitive Type II (*t*-test, *P* < 0.05, *T* = 2.02) and non-canal neurons (*t*-test, *P* < 0.0005, *T* = 3.95) were significantly higher than that of the non-sound sensitive neurons (Figure 4B). A correlation analysis was performed to examine the relationship between sensitivity to head rotation and sound sensitivity for Type I and Type II sound sensitive neurons (Figure 5). The Type I neurons’ CPEs were weakly inversely correlated with their gains (*R* = 0.35, *P* = 0.022, open symbols). There was no significant correlation for the Type II neurons. Histology showed that sound sensitive neurons were distributed through the vestibular nuclei. There were no clusters of sound sensitive neurons or non-sound sensitive neurons.

#### Tone Burst-Evoked Responses of Vestibular Nucleus Neurons

**FIGURE 6:**
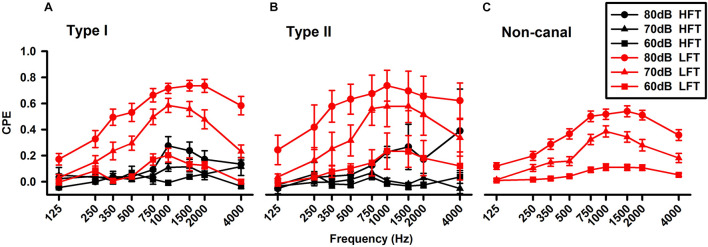
Averaged frequency tuning of the vestibular nucleus neurons. **(A)** Type I vestibular neurons. **(B)** Type II vestibular neurons. **(C)** Non-canal vestibular neurons. The sound sensitive neurons were further classified as LFT (low frequency threshold, red lines) and HFT (high frequency threshold, black lines) neurons. The Type I and II neurons, which included canal-only and canal-otolith convergent neurons, exhibited both HFT and LFT responses, while the non-canal neurons (otolith only) exhibited only LFT responses.

Tone-evoked responses were examined in 208 neurons. Similar to the click-evoked responses, tone evoked more responses in the non-canal neurons (49%) than in the Type I neurons (31%, Chi-square test, *P* < 0.05) and the Type II units (25%, Chi-square test, *P* < 0.001; Table 1). The vestibular nucleus neurons exhibited well-defined tuning curves, which exhibited peak responses at around 1,500 Hz at 70 and 80 dB (Figure 6). An earlier study of vestibular afferent tuning curves revealed that an important difference between the otolith afferents and the canal afferents is that low frequency tones of 350 Hz can activate the otolith afferents, but not the canal afferents (Zhu et al., 2011a). Thus, if a vestibular nucleus neuron only receives inputs from canal afferents, it would not be activated by low frequency tones. However, if it receives inputs from the otolith afferents, it would be activated by low frequency tones. In this study, we classified the neurons that exhibited significant responses to tones of 350 Hz (CPEs > 0.1) as low frequency threshold neuron (LFT, red) and the neurons that did not exhibit responses to tones of 350 Hz as high frequency threshold neurons (HFT, black). The Type I and Type II neurons, which included the canal-only and the otolith-canal convergent neurons, had both HFT and LFT responses. However, the non-canal neurons, which were the otolith-only neurons, had only LFT responses. Consistent with the assumption of their inputs from both the otolith afferents and canal afferents, the Type I and Type II LFT neurons exhibited larger tone-evoked responses than those of the non-canal neurons. One way ANOVA analysis revealed that the peak responses of non-canal neurons were significant lower than those of the Type I neurons (*P* < 0.05, 1,500 Hz, 80 dB SL). It is important to note that the HFT/LFT classification is solely based on tone-evoked responses and their anatomical connections to the vestibular afferents need to be confirmed by future studies.

### Sound Sensitivity of the Abducens Neurons

**FIGURE 7:**
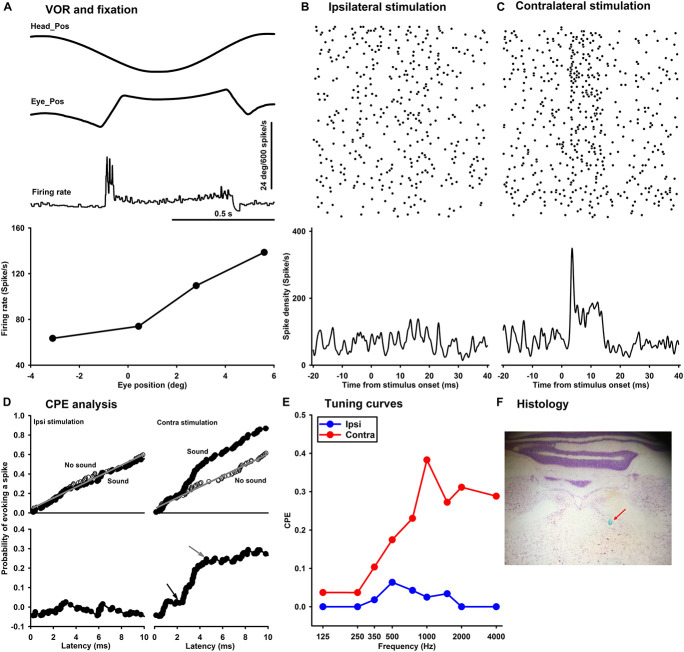
Sound-evoked responses in a representative abducens neuron in an awake rat. **(A)** Discharge activities during eye movements in response to head rotation (**A**, top and middle panels) and fixations (**A**, lower panel). Raster of the abducens neuron in response to 1,000 Hz tone bursts delivered into the ipsilateral ear **(B)** or the contralateral ear **(C)**. **(D)** CPE analysis of tone-evoked responses in the abducens neuron. **(E)** Tuning curves of the abducens neurons for ipsilateral (blue) and contralateral (red) ear stimulation. **(F)** Histological verification of a recording site in the abducens nucleus.

**FIGURE 8:**
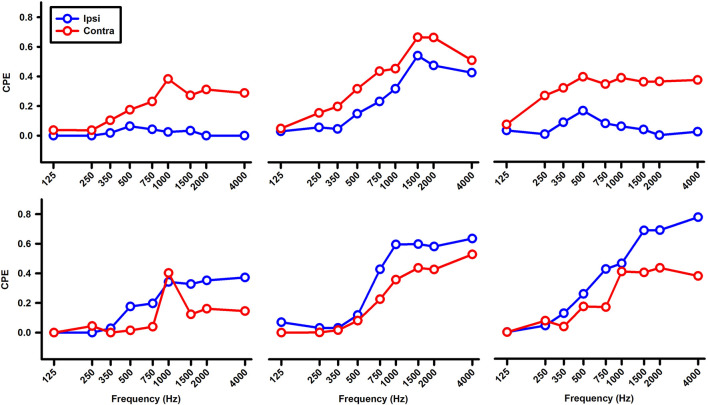
Tuning curves of six abducens neurons for ipsilateral (blue) and contralateral (red) ear tone stimulation.

In a pilot study, we examined the responses of abducens neurons to sound stimulation in 19 rats under isoflurane anesthesia. Among 90 abducens neurons tested with clicks, only two neurons were activated by the ipsilateral ear stimulation. Among 50 neurons tested with tone bursts, only one neuron was activated by sound stimulation to either the ipsilateral or contralateral ear. Thus, different from the vestibular afferents and the vestibular nuclei, sound activation of abducens neurons could be suppressed by anesthesia. In the current study, abducens neuron responses to tone bursts or clicks were studied in awake rats. Figure 7 shows typical responses of an abducens neuron during fixations and horizontal eye movements evoked by head rotation. The neuron exhibited a burst during a saccade in its on-direction and a pause during a saccade in its off-direction (Figure 7A, top and middle panels). The abducens neuron firing rate was linearly related to horizontal eye position with a sensitivity of 8.9 spike/s/deg (Figure 7A, lower panel). Figures 7B,C show rasters and spike densities of the neuron in response to tone bursts (1,000 Hz, 8 ms plateau, 1 ms rise/fall, 80 dB SL) delivered to the ipsilateral or contralateral ear, respectively. CPEs were computed to quantify the neuron’s sound sensitivity (Figure 7D). Figure 7E shows the tuning curves of the abducens neuron to tones delivered into the ipsilateral and contralateral ears. While this neuron exhibited responses to tone bursts in both ears, its responses to the contralateral stimulation, which was mediated by the horizontal canal inputs, were much larger than that to the ipsilateral stimulation, which was mediated by the utricule inputs (Uchino et al., 1996). Among 18 abducens neurons studied, seven of them exhibited well-defined responses to tone bursts or clicks. Four neurons exhibited excitatory response to both ipsilateral and contralateral sound, indicating convergence of inputs from both the canal and the utricular afferents. Two neurons only responded to contralateral tones, but not to the ipsilateral tones, indicating inputs only from the canal afferents. One neuron exhibited responses only to the ipsilateral sound, indicating inputs from the otolith afferents. Similar to the vestibular nucleus neurons, the abducens neurons exhibited both LFT and HFT responses to tone stimulation (Figure 8).

On average, the click-evoked responses of the abducens neurons had a latency of 2.95 ± 0.45 ms and CPE of 0.162 ± 0.05 to ipsilateral stimulation, and a latency of 3.09 ± 0.68 ms and CPE of 0.157 ± 0.05 to contralateral stimulation. Similar to the latencies of click-evoked responses in the vestibular nucleus neurons (2nd order VOR neurons), which were longer than that in the vestibular afferents (1st order VOR neurons) (∼0.7 ms) (Zhu et al., 2011b), the latencies of click-evoked responses in the abducens neurons (3rd order VOR neurons) were longer than that in the vestibular nucleus neurons (∼1.5 ms).

### Tone Burst-Evoked Eye Movements

**FIGURE 9:**
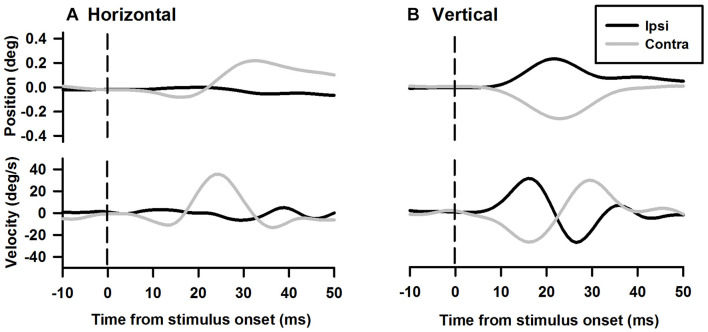
Representative eye movement responses to tone bursts of 1,000 Hz. **(A)** Tone-evoked horizontal eye movement responses in position (*upper panel*) and velocity (*lower panel*). **(B)** Tone-evoked vertical eye movement responses in position and velocity (*lower panel*).

**FIGURE 10:**
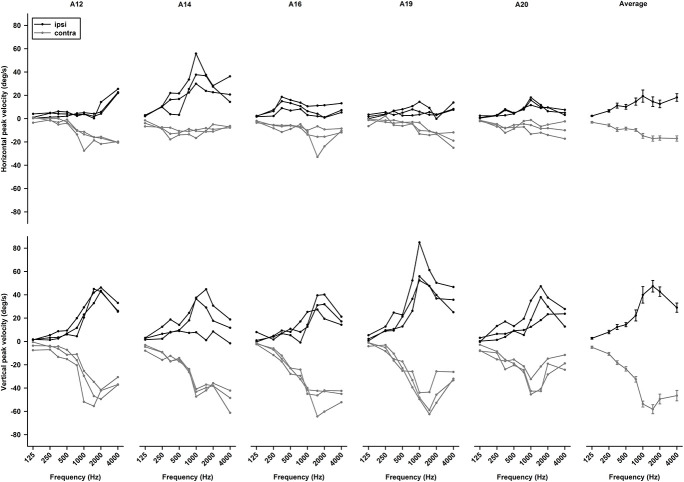
Repeatability of tone bursts-evoked eye movements. *Upper panels* are horizontal eye movement tuning curves of five rats obtained in three consecutive days. *Lower panels* are vertical eye movement tuning curves of rats obtained in three consecutive days. The last *right panels* are averaged tuning curves from the five rats.

Tone-evoked eye movements were studied in the left eye of five female LE rats. Figure 9 shows typical changes in horizontal and vertical eye position and velocity to tone bursts of 1,000 Hz at 80 dB SL delivered to the ipsilateral (black lines) or contralateral ear (gray lines). In the horizontal direction, tone bursts evoked small eye movements at a latency of ∼4.4 ms, which moved toward the nose for the ipsilateral stimulation, and toward the ear for the contralateral stimulation (Figure 9A). In the vertical direction, tone bursts evoked upward eye movements for the ipsilateral stimulation and downward eye movements for the contralateral stimulation (Figure 9B). The tone-evoked eye movements exhibited a bell shape response in position with a peak latency of ∼20.7 ms and a biphasic response in velocity with a first peak latency of ∼15.9 ms. To quantitatively examine how tone-evoked eye movements are dependent on tone frequency, tuning curves were compiled using the first peak of eye velocity response. Figure 10 shows tuning curves in the horizontal and vertical directions for the ipsilateral (black lines) and contralateral (gray lines) stimulation in the group of rats. Each rat was tested for three consecutive days to examine repeatability of the tone-evoked eye movements (Figure 10, three black lines and three gray lines in each panel). While there were individual variability and day-to-day variability, tone bursts consistently evoked well-defined tuning curves for the vertical eye movements with peak frequencies at ∼1,500 Hz. The tone-evoked horizontal eye movements, however, not only were smaller than tone-evoked vertical eye movements (horizontal peak velocity: 13.7 deg/s, vertical peak velocity: 36.8 deg/s), but also increased with tone frequencies up to 4,000 Hz. An important feature of the tone-evoked eye movements is that tone bursts with 350 Hz or lower evoked well-defined eye movements, consistent with convergence of canal and otolith contributions on the abducens neurons.

**FIGURE 11:**
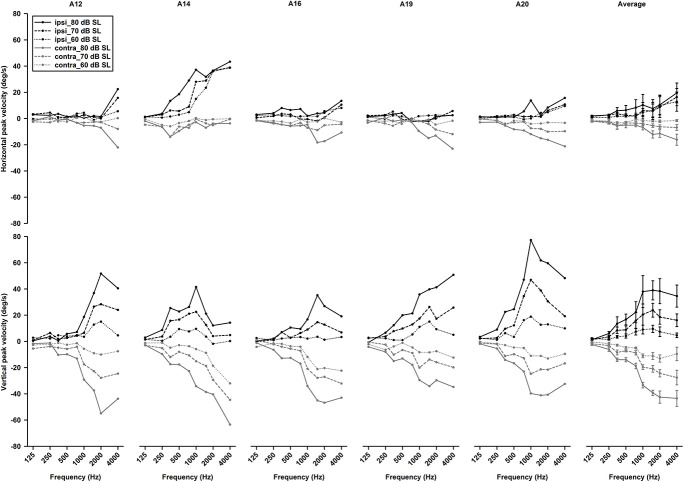
Effects of intensity on tone bursts-evoked horizontal (*upper panels*) and vertical (*lower panels*) eye movements in five rats (the left five panels). The last right panels are the averaged tuning curves of the five rats.

**FIGURE 12:**
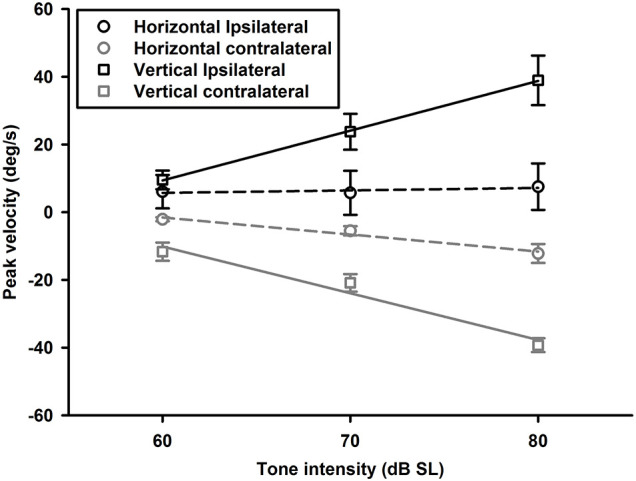
Averaged peak velocities of 1,500 Hz tone burst-evoked horizontal (circles) and vertical (squares) eye movements as a function of tone intensity. Black lines and symbols are for contralateral ear stimulation, and gray lines and symbols are for ipsilateral ear stimulation.

**FIGURE 13:**
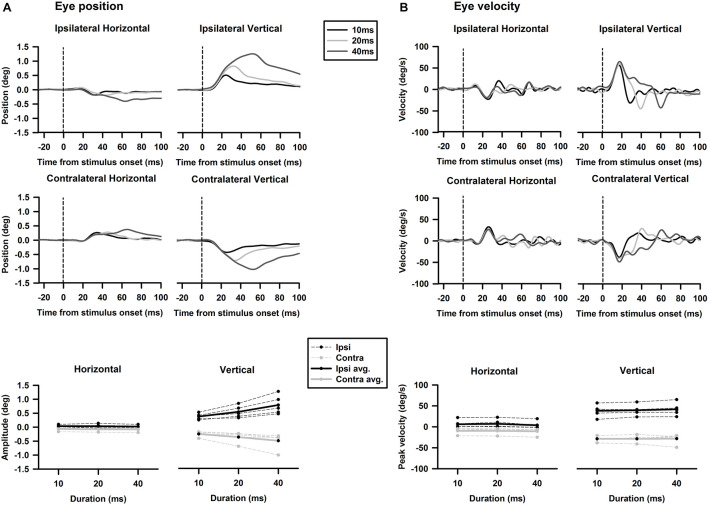
Effects of duration on tone-bursts-evoked horizontal and vertical eye movements. **(A)** Tone-evoked changes in eye position. **(B)** Tone-evoked changes in eye velocity.

To examine effects of intensity on tone-evoked eye movements, we compiled the tuning curves for tone bursts at three intensities (60, 70, 80 dB SL) (Figure 11). To quantitatively assess effects of intensity on horizontal and vertical eye movements, peak velocities of 1,500 Hz tone-evoked eye movements were plotted as a function of intensity for ipsilateral (black lines) and contralateral (gray lines) stimulation (Figure 12). While sound-evoked vertical eye movements increased with tone intensity for either ipsilateral (slope: 1.47 deg/s/dB, *R*^2^ = 0.99) or contralateral (slope: -1.38 deg/s/dB, *R*^2^ = 0.93) stimulation, sound-evoked horizontal eye movements increased with tone intensity only for contralateral (slope: -0.5 deg/s/dB, *R*^2^ = 0.93) stimulation, but not for ipsilateral (slope: 0.07 deg/s/dB, *R*^2^ = 0.15) stimulation. Figure 13 further showed how tone-evoked eye movements were dependent on tone duration. When tone duration was increased, tone-evoked changes in eye position were increased only in the vertical direction. The peak velocities in the horizontal and vertical directions, however, were not changed.

## Discussion

In the present study, we extended the early studies of sound-activation of the vestibular afferents and further examined how sound activates the other components of the VOR pathways, i.e., the vestibular nucleus neurons, the abducens neurons and finally the eye movements. The main finding is that sound-activation of the vestibular nucleus neurons, abducens neurons, and eye movements reflects summation of sound activation of the canal and otolith afferents. These results provide insight into understanding the neural mechanisms underlying the VEMPs and developing discriminative VEMP protocols utilizing tone bursts-evoked eye movements in animal models and in humans. Since eye movements are controlled by interactions of agonist-antagonist pairs of extraocular muscles, sound-evoked eye movements need to be interpreted as interactions of sound activation of multiple VOR pathways.

### Sound-Activation of the Vestibular Nucleus Neurons

Click-evoked responses in the vestibular nuclei were reported in anesthetized guinea pigs (Murofushi et al., 1996). In the study, some of the sound sensitive vestibular nucleus neurons were found to respond to head tilt, indicating their inputs from the otolith afferents. The present study extended the previous study by quantitatively examining tone burst-evoked responses in the rat vestibular nucleus neurons, which were further classified into three categories based on their responses to head rotation, i.e., the Type I, Type II, and non-canal neurons (Scudder and Fuchs, 1992). The Type I and Type II neurons exhibited increased and decreased firing rates to ipsilateral head rotation, respectively. The non-canal neurons exhibited no modulation to head rotation. While the non-canal neurons likely only receive otolith inputs, the Type I and Type II neurons may consist of neurons that only receive canal inputs and neurons that also receive otolith inputs (Uchino et al., 2005). As shown in our earlier studies of tone-evoked activation of the vestibular afferents, the canal and otolith afferents exhibit distinct characteristics to tone frequency (Zhu et al., 2011a). First, the canal afferents exhibited no responses to tones with frequencies at 350 Hz or lower, even at the highest intensity tested (80 dB SL). However, the otolith afferents exhibited well-defined responses to tones of 350 Hz, even at the moderate intensity (70 dB SL). Second, tones of 1,500 Hz activate both canal and otolith afferents at intensities of 70 dB or higher, but only the otolith afferents at the intensity of 60 dB SL. For the Type I/II neurons that only receive the canal inputs, they would be expected to exhibit responses to tones with frequencies higher than 350 Hz, i.e., high frequency threshold (HFT). For the Type I/Type II neurons that receive both the canal and otolith inputs, they would be expected to exhibit responses to tones with frequencies at 350 Hz or lower, i.e., low frequency threshold (LFT). Indeed, the non-canal neurons were all responsive to low frequency tones (i.e., HFT neurons), indicating their inputs from the otolith afferents. Similarly, the Type I/II neurons have both LFT neurons and HFT neurons, indicating some of them receiving inputs from both the canal afferents and the otolith afferents. Furthermore, the LFT Type I/II neurons exhibited larger tone-evoked responses than the LFT non-canal neurons, consistent with the assumption of their converged inputs from the canal and otolith afferents. These results suggest that in the vestibular nuclei, the sound-evoked afferent responses are primarily carried by the otolith only neurons and the canal/otolith convergence neurons.

### Sound Activation of the Abducens Neurons

The abducens nucleus has the final neurons of the VOR pathways. On one hand, they receive canal inputs from the VOR interneurons in the contralateral vestibular nuclei and otolith inputs from the ipsilateral utricular afferents. On the other hand, abducens neurons innervate the lateral rectus of the ipsilateral eye via its motoneurons and the medial rectus of the contralateral eye via its internuclear neurons (Highstein and Baker, 1978). Thus, for the eye ipsilateral to the stimulated ear, its abducens neurons receive utricular inputs either directly from the ipsilateral utricular afferents or indirectly from the secondary vestibular nucleus neurons that receive utricular afferent inputs (Uchino et al., 1996). For the eye contralateral to the stimulated ear, its abducens neurons receive inputs from the canal afferents from the stimulated side. It is important to note that some vestibular nucleus neurons receive inputs from both the canal afferents and the otolith afferents, such as those Type I and Type II vestibular nucleus neurons that exhibit low frequency threshold responses. As a result of the convergence, the abducens neurons in the contralateral VOR pathways are activated by sound activation of the otolith afferents, such as the neurons in Figures 7, 8. While click-evoked responses have been studied in monkey abducens neurons (Zhou et al., 2007; Xu et al., 2009), to the best of our knowledge, this was the first study that examined tone burst-evoked responses in the abducens neurons in awake rats. The results show that sound activation of the vestibular afferents results in activations of abducens neurons in the ipsilateral and contralateral sides, which contribute to the sound-evoked eye movements measured by a video-based eye tracker (Figures 9–13).

### Tone Bursts-Evoked Eye Movements

The sound-evoked responses in the vestibular nucleus neurons and abducens neurons suggest that sound activation of the vestibular afferents, the first neuron in the three-neuron arc of the VOR pathways, results in a robust activation of the second and third order neurons of the circuits, which is expected to generate measurable tone-evoked eye movements. Indeed, using a video-based eye tracker, we observed well-defined tone-evoked eye movements, which were dependent on frequency, duration, and intensity. To interpret the sound-evoked horizontal and vertical eye movements, it is important to note that eye movements are generated not by actions of a single extraocular muscle, but by actions of agonist-antagonist pairs of the extraocular muscles. For example, the horizontal eye movements of the left eye are determined by the actions of the medial rectus and the lateral rectus, which are innervated by motoneurons in the oculomotor nuclei and abducens nuclei, respectively. As shown in Figures 11, 12, the effects of intensity on the horizontal eye movements evoked by the ipsilateral stimulation were quite different from that evoked by the contralateral stimulation. Whereas the contralateral stimulation-evoked eye movements were increased with tone intensity, the ipsilateral stimulation-evoked eye movements exhibited little changes as intensity were increased (Figure 12). The results suggest that the ipsilateral stimulation evoked two types of eye movements that were in opposite directions. One type of eye movement was generated by activation of the utricular-abducens pathways that moved the left eye to the left, and the other type of eye movement was generated by the canal-oculomotor pathways (via the abducens internuclear neurons) that moved the left eye to the right. The observed eye movements was the summation of the two types of eye movements that were in opposite directions. Since the canal pathways generated larger activation of the medial rectus, the eye moved to the right with reduced amplitudes. Thus, although the amplitudes of both types of eye movements increased with tone intensity, the summed results exhibited little changes with different intensities.

Sound evoked different eye movements in the horizontal and vertical directions. First, sound- evoked horizontal movements in the two eyes were in the same direction, i.e., away from the stimulated ear, and sound evoked vertical eye movements in the two eyes were in opposite directions, i.e., upward in the ipsilateral eye and downward in the contralateral eye. Second, the sound-evoked vertical eye movements were much larger than the horizontal eye movements. In fact, the vertical eye movement were about three times larger than the horizontal eye movements. Third, low frequency tone bursts (350 Hz) evoked minimal eye movement in the horizontal direction, but evoked robust responses in the vertical direction in both eyes. As shown in Table 2, the vertical eye movements evoked by ipsilateral stimulation are the summed results of four VOR pathways, i.e., the anterior canal-superior rectus and the utricule-superior rectus pathways that move the eye upward, and the posterior canal-superior oblique muscle and the utricle-superior oblique muscle pathways that move the eye downward (McCrea et al., 1987a,b; Uchino et al., 1996). In contrast to the superior rectus that is the main extraocular muscle that rotates the eye upward, the superior oblique muscle rotates the eye partially downward and partially clockwise. If there is a similar sound activation in the two extraocular muscles, the superior rectus action is the dominant force and the eye rotates upward. Similar interpretation applies to the tone-evoked downward eye movement in the eye contralateral to the stimulated ear, which are the summed results of four VOR pathways (Table 2), i.e., the posterior canal-inferior rectus and the utricule-inferior rectus pathways that move the eye downward, and the anterior canal-inferior oblique muscle and the utricle-inferior oblique muscle pathways that move the eye upward. In this case, the inferior rectus action is the dominant force that rotates the contralateral eye downward. The vertical eye movement responses to low frequency tones are consistent with the contributions from sound activation of the utricular pathways.

**TABLE 2:** Vestibulo-ocular reflex (VOR) pathways that connect vestibular end organs to extraocular muscles of the left eye.

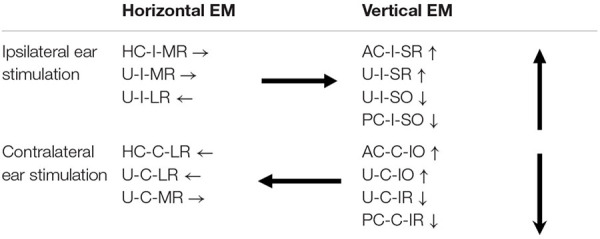

*HC, horizontal canal; AC, anterior canal; PC, posterior canal; U, utricule; LR, lateral rectus; MR, medial rectus; SR, superior rectus; SO, superior oblique; IR, inferior rectus; IO, inferior oblique; I, ipsilateral; C, contralateral; →, rightward eye movement; ←, leftward eye movement; ↑, upward eye movement; ↓, downward eye movement.*

Our earlier studies showed that clicks and tone bursts evoked well-defined eye movements in monkeys (Zhou et al., 2004, 2007; Xu et al., 2009). It is worth comparing sound-evoked eye movements in rats and monkeys. On one hand, sound evokes conjugate horizontal eye movements in both rats and monkeys. On the other hand, there are notable differences in sound-evoked responses in the two animal models. First, sound evoked disjunctive vertical eye movements in all five rats tested, but it evoked conjugate vertical eye movements in one monkey and disjunctive vertical eye movements in another monkey. Second, the amplitudes of tone-evoked vertical eye movements in rats are about 20 times larger than that in monkeys. Third, the tone bursts-evoked horizontal eye movements in the eye ipsilateral to the stimulated ear were increased with sound intensity in monkeys, but not in rats. In humans, the click-evoked VOR responses have been studied in normal subjects and in patients with superior canal dehiscence (SCD). Although clicks evoked well-defined eye movements in SCD patients, the studies detected minimal eye movements in normal humans (Aw et al., 2006).

### Summary and Future Studies

In this study, we provided evidence that sound activation of the vestibular afferents further leads to activation of the VOR interneurons and motoneurons, which produces sound-evoked eye movements that can be measured by a video-based eye tracker. Consistent with convergence of canal and otolith inputs on the vestibular nucleus neurons, we showed that the vestibular nucleus neurons exhibited well-defined tone bursts-evoked activation that reflected activation of both the canal and otolith afferents. This convergence of sound activation of the canal and otolith afferents were further observed in the abducens neurons and the tone burst-evoked eye movements. An interesting finding of the study is that tone bursts-evoked robust eye movements in rats were much larger than that reported in monkeys and humans, which can be measured by a video-based eye tracker. Since the tone bursts-evoked eye movements reflect sound-activation of both the canals and the otoliths, it can be developed into an important biomarker for assessing unilateral vestibular functions in diseased conditions in animal models. Furthermore, it calls for reexamination of the protocols used in studies of sound-evoked eye movements in humans. For example, instead of using clicks that primarily contains high frequency components, our results suggest that tone bursts of various frequencies should be employed and binocular eye movements should be measured. Sound activation of both the canals and otoliths needs to be taken into consideration when interpreting sound-evoked eye movements in humans.

## Data Availability Statement

The original contributions presented in the study are included in the article/supplementary material, further inquiries can be directed to the corresponding authors.

## Ethics Statement

The animal study was reviewed and approved by IACUC of University of Mississippi Medical Center.

## Author Contributions

HZ and WZ designed the research. TC, JH, YY, XT, CZ, YX, AA, JA, WM, MD, TR, MZ, HZ, and WZ performed data acquisition and data analysis. HZ and WZ wrote the manuscript. All authors contributed to the article and approved the submitted version.

## Conflict of Interest

The authors declare that the research was conducted in the absence of any commercial or financial relationships that could be construed as a potential conflict of interest.

## Publisher’s Note

All claims expressed in this article are solely those of the authors and do not necessarily represent those of their affiliated organizations, or those of the publisher, the editors and the reviewers. Any product that may be evaluated in this article, or claim that may be made by its manufacturer, is not guaranteed or endorsed by the publisher.
